# Analysis of the Electricity Consumption in Municipal Wastewater Treatment Plants in Northeast China in Terms of Wastewater Characteristics

**DOI:** 10.3390/ijerph192114398

**Published:** 2022-11-03

**Authors:** Xuege Wang, Yanhong Dong, Shuang Yu, Guangyi Mu, Hong Qu, Zhuan Li, Dejun Bian

**Affiliations:** 1 Jilin Provincial Key Laboratory of Municipal Wastewater Treatment, Changchun Institute of Technology, Changchun 130012, China; 2 China Northeast Municipal Engineering Design and Research Institute Co., Ltd., Changchun 130021, China

**Keywords:** sewage treatment plant, influent indicator, effluent indicator, electricity consumption, statistical analysis

## Abstract

A municipal wastewater treatment plant plays an important role in treating urban sewage and reducing the quantity of pollutants discharged into rivers. However, the energy consumption of the municipal wastewater treatment industry is large. High energy consumption indirectly produces ecological damage, accelerates the energy crisis, and increases carbon emissions. For energy conservation and emission reduction in wastewater treatment plants, it is first necessary to identify the main factors influencing energy consumption. Electricity consumption accounts for more than 80% of the energy consumption of wastewater treatment plants. Wastewater quantity and wastewater quality have become the key influencing factors of energy conservation and consumption reduction in wastewater treatment plants. In this study, a municipal wastewater treatment plant in Northeast China was selected as the research object, and the measured data, such as air temperature, wastewater quantity, wastewater quality, and electricity consumption of the plant from 2017 to 2020 were statistically analyzed to explore the influences of temperature and wastewater quantity and wastewater quality indicators of influent and effluent on energy consumption. Firstly, the range of influent quantity in the wastewater treatment plant was large. The influent quantity in summer was high because some rainwater entered the sewage treatment plant. In winter, average daily electricity consumption (ADEC) was higher than that in summer. The relationship between ADEC and the wastewater quantity showed a positive correlation, and ADEC slowly increased with the increase in wastewater quantity. Electricity consumption per unit of wastewater (UEC) was negatively correlated with the wastewater quantity, but the correction coefficient in winter was larger than that in summer. Secondly, the ranges of chemical oxygen demand (COD_Cr_) and ammonia nitrogen in influent were large, and the ranges of COD_Cr_ and ammonia nitrogen in effluent were small. Influent COD_Cr_ concentration was negatively correlated with influent ammonia nitrogen concentration. ADEC increased slightly with the increase in influent COD_Cr_ concentration. In winter, the increasing trend of ADEC with the influent COD_Cr_ concentration was higher than that in the summer. The increasing trend of UEC with the increase in influent COD concentration in summer was more significant than that in winter. Thirdly, influent COD_Cr_ in 11.6% of the samples exceeded the corresponding designed value, and influent ammonia nitrogen concentration in 41.4% of the samples exceeded the corresponding designed value. Effluent COD_Cr_ in 10.6% of the samples exceeded the First Level Class B standard in “Discharge Standard of Pollutants for Municipal Wastewater Treatment Plants (GB18918-2002)”, and unqualified COD_Cr_ in 94% of the effluent samples was ascribed to the unqualified ammonia nitrogen concentration in the influent samples. The electricity consumption level under abnormal conditions was higher than that under normal conditions. Fourthly, ADEC was positively correlated with the average daily COD_Cr_ reduction. The correction coefficient of ADEC with average daily COD_Cr_ reduction was greater in winter than that in summer. Fifthly, the average electricity consumption per unit of wastewater was close to the national average energy consumption, displaying the characteristics of high energy consumption in winter and low energy consumption in summer. The correlation analysis results of unit electricity consumption and temperature showed that when it was below 0 °C, the lower the temperature, the higher the electricity consumption. In Northeast China, the influences of seasons and temperatures on the electricity consumption of sewage plants were obvious. Accordingly, it is necessary to implement the diversion of rainwater and sewage, reduce the discharge of unqualified wastewater from enterprises, and take thermal insulation measures in winter. In addition, activated sludge microorganisms suitable for a low temperature area and the optimal scheduling of sewage pipe networks can also improve the operation and management of sewage treatment plants.

## 1. Introduction

Due to urban development, the production of industrial wastewater and domestic sewage has increased yearly. An urban sewage treatment plant is significant in treating urban sewage and reducing the quantity of pollutants discharged into rivers. The stable operation of urban sewage treatment plants is important in improving water quality. However, the municipal sewage treatment process is actually a process of energy consumption and pollution transfer [1]. The energy consumption of a sewage treatment plant mainly includes direct energy consumption and indirect energy consumption. Direct energy consumption is the electricity consumption in the operation of pumps, aeration, and oxygenation equipment, and indirect energy consumption mainly involves chemical phosphorus removal and chemical agents. Electricity consumption accounts for more than 80% of the energy consumption of wastewater treatment plants. High energy consumption leads to high cost [2], both of which are also bottlenecks of sewage treatment plants [3]. In addition, high energy consumption also indirectly produces ecological damage, accelerates the energy crisis, and increases carbon emissions [4,5,6,7]. A reasonable assessment of energy consumption is significant for energy conservation and emission reduction in sewage treatment plants [8,9,10,11,12]. The key to reasonable energy consumption evaluation is to identify the main influencing factors of energy consumption.

Previous studies on sewage treatment plants primarily focused on the upgrading of sewage treatment plants [13,14], sewage treatment processes [15,16,17,18], the enhancement of nitrogen and phosphorus removal [19,20,21,22,23,24,25,26,27,28], and treatment costs [29,30,31,32]. The studies on the energy consumption of sewage treatment plants were basically concentrated on statistical analysis, and the corresponding changes in energy consumption with wastewater quantity, wastewater quality, and treatment process [33,34,35,36,37]. These studies were based on annual average statistics and significant for the control of energy consumption of all sewage treatment plants in the country, but the data of a single sewage treatment plant were not explored. In addition, the indicators of the wastewater quantity and wastewater quality of a sewage treatment plant are changeable and it is difficult to explore the variations from the annual average data.

Electricity consumption is closely related to some factors such as equipment type, treatment process, wastewater quantity, pollutant concentration, operation parameters, and other factors of sewage treatment plants. Wastewater quantity and wastewater quality are the key influencing factors for energy conservation and consumption reduction in existing sewage treatment plants under stable operation conditions. The studies on the overall changes of the indicators of wastewater quality and quantity of the influent and effluent of a sewage treatment plant and the correlation with energy consumption are of great significance for saving the operation cost, improving effluent indicators, and reducing the energy consumption of sewage treatment plants [38,39,40]. Hence, in this study, with an urban sewage treatment plant in Northeast China as an example, the variations of wastewater quantity, wastewater quality, temperature, and electricity consumption indicators of the plant from 2017 to 2020 were analyzed to identify the primary factors of energy consumption. The study provides the basis for the operation, management, design, energy conservation, and consumption reduction in sewage treatment plants in the cities in Northeast China.

## 2. Materials and Methods

**Figure 1:**
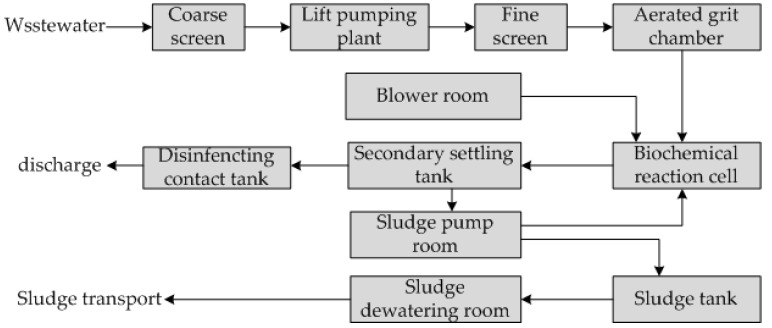
Process flowchart of a sewage treatment plant in Northeast China.

A municipal sewage treatment plant in Northeast China was selected as the target in the study. The plant has a daily treatment capacity of 15 × 10^4^ m^3^/d and adopts an anaerobic–anoxic–oxic (A^2^O) treatment process. Figure 1 demonstrates the process flow. The designed influent indicators are provided as follows: 420 mg/L of chemical oxygen demand (COD_Cr_) and 30 mg/L of ammonia nitrogen (NH_3_-N). Effluent met with the First Level Class B Standard in “Discharge Standard of Pollutants for Municipal Wastewater Treatment Plants (GB18918-2002)”.

In this study, the measured data of the influent and effluent of the sewage treatment plant from 2017 to 2020, including wastewater quantity, wastewater quality, and electricity consumption, were analyzed with the multivariate statistical analysis methods [41,42]. The SPSS software was used for the statistical analyses of wastewater quality and unit electricity consumption, primarily including frequency analysis, monthly variation analysis, and regression analysis. Table 1 demonstrates the statistical results of various indicators of a sewage treatment plant in Northeast China.

The electricity consumption per unit wastewater quantity (UEC) and average daily COD_cr_ reduction were estimated as follows:

$$\mathrm{UEC}=\frac{d}{q}$$

$$DCR=\left(COD_{i}-COD_{e}\right)\times q$$

where UEC is the electricity consumption per unit wastewater quantity; *d* is the average daily electricity consumption (ADEC); *q* is the wastewater quantity; *DCR* is the average daily COD_cr_ reduction; $COD_{i}$ is the influent COD_cr_ concentration; and $COD_{e}$ is the effluent COD_cr_ concentration.

## 3. Results

### 3.1. Characteristics of Wastewater Quantity Indicators of the Sewage Treatment Plant

**Figure 2:**
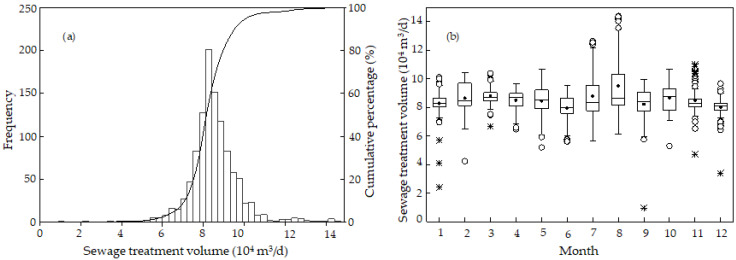
(**a**) Probability and (**b**) monthly distributions of daily average sewage treatment volume. In (**b**), the five horizontal lines in each box represent the five percentile points. The horizontal line on the lower surface represents the lower value; the lower end of the box represents the 25th percentile value (p25); the line segment in the box represents the median value; the top of the box represents the 75th percentile value (p75); the top horizontal line represents the largest value. The solid circle (•) represents the average. Outliers are marked by a hollow point (°) and extreme points are marked by an asterisk (*).

Figure 2 shows the probability, annual, and monthly distributions of the average daily sewage treatment quantity. Figure 2a indicates that the wastewater treatment quantity of the sewage treatment plant was between 0.9 × 10^4^ and 14.4 × 10^4^ m^3^/d and below the design value of 15 × 10^4^ m^3^/d. The average daily sewage treatment quantity was in the range of 5.5~11 × 10^4^ m^3^/d, in which the cumulative distribution probability was above 98%. The daily sewage treatment quantity was mainly concentrated in the range of 8~10 × 10^4^ m^3^/d, in which the cumulative distribution probability reached 70%. The cumulative distribution probability of the sewage treatment quantity between 0 and 10.3 × 10^4^ m^3^/d was 95%. The median value decreased from January to December and was concentrated between 8 × 10^4^ and 9 × 10^4^ m^3^/d (Figure 2b). The median value was the lowest in June and December and the highest in October. The monthly change was low in January, March, November, and December and high in July and August. The results might be interpreted as follows. In July and August in summer, the weather is hot, and the water consumption of residents and corresponding wastewater also increase. In addition, the city has not yet implemented a complete rainwater-sewage diversion system, and the summer rainfall in Northeast China is concentrated in July and August, thus, resulting in some rainwater entering the sewage treatment plant [43,44,45]. The monthly change from p25 to p75 was more significant in February, May, July, August, and October due to the high water consumption in the Spring Festival, summer, and long holidays in May and October [43].

### 3.2. Variations of Wastewater Quality Indicators of Influent of the Sewage Treatment Plant

#### 3.2.1. Influent COD_Cr_ Concentration

**Figure 3:**
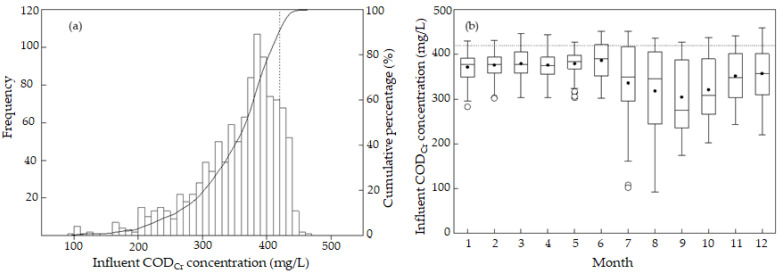
(**a**) Probability distribution and (**b**) monthly distribution of influent COD_Cr_ concentration. The solid circle (•) represents the average. Outliers are marked by a hollow point (°).

Figure 3 shows the monthly distribution and probability distribution of the daily average concentration of COD_Cr_ in the influent of the sewage treatment plant. The minimum and maximum values of daily average COD_Cr_ in influent were, respectively, 92 mg/L and 460 mg/L, indicating the large range of influent COD_Cr_ (Figure 3a). The daily average COD_Cr_ was mainly distributed in the range of 350 to 450 mg/L, in which the cumulative distribution probability was 62%. The most common COD_Cr_ was 390 mg/L. When influent COD_Cr_ was between 0 and 300 mg/L, the cumulative distribution probability was 17.5%. When influent COD_Cr_ was 420 mg/L, the cumulative distribution probability was 88.4%, indicating that 11.6% of the COD_Cr_ data of the influent exceeded the designed influent COD_Cr_. The maximum influent COD_Cr_ was 1.09 times of the designed COD_Cr_, 420 mg/L.

Influent COD_Cr_ was the highest in December; lower (less than 300 mg/L) in July, August, September, and October; and the lowest in August (Figure 3b). From January to May, the range of influent COD_Cr_ was small, and the median value of influent COD_Cr_ was constant at approximately 380 mg/L. The variation range of influent COD_Cr_ was small from January to May, significant from July to December, and the most significant in August. The change of the median value was also significant in August, whereas the median value was the lowest in September and only 275 mg/L. The median value in October was 308 mg/L. The p25 value was the lowest in September, followed by August. Influent COD_Cr_ was also the lowest in August. The data indicated that in summer (July, August, and September), influent COD_Cr_ was low. Influent COD_Cr_ never exceeded the designed value in each month (Figure 3b). In particular, the p75 value in June exceeded the designed index of 420 mg/L.

#### 3.2.2. Influent Ammonia Nitrogen

**Figure 4:**
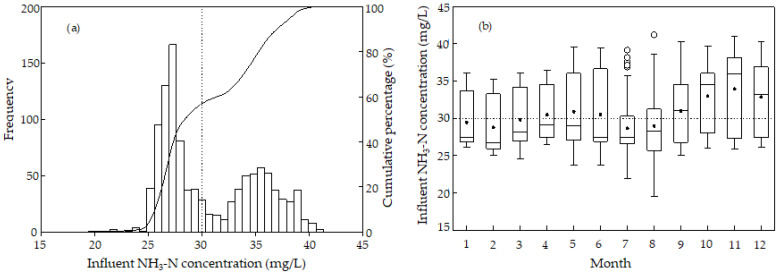
(**a**) Probability distribution and (**b**) monthly distribution of influent NH_3_-N concentration. The solid circle (•) represents the average. Outliers are marked by a hollow point (°).

Figure 4 shows the monthly distribution and probability distribution of the daily average concentration of ammonia nitrogen in influent in the sewage treatment plant. Figure 4a shows that the influent ammonia nitrogen concentration does not follow a normal distribution. The range of influent ammonia nitrogen concentration was 19.55 to 41.2 mg/L and could be roughly divided into two concentrated ranges: 25~30 mg/L with the concentration corresponding to the highest frequency (27 mg/L), and 30~40 mg/L with the concentration corresponding to the highest frequency (36 mg/L). Among all the determined influent ammonia nitrogen concentrations, 58.6% of the data were below the designed value, 30 mg/L. In other words, 41.4% of the data measured exceeded the designed value. The maximum influent ammonia nitrogen concentration was 1.37 times of the designed value, 30 mg/L.

The influent ammonia nitrogen concentration was the lowest in August and the highest in November (Figure 4b). In July and August, the influent ammonia nitrogen concentration range was small, while the range in other months was large. The median value of influent ammonia nitrogen concentration from January to August was between 27 and 29 mg/L, whereas the median value from September to December was between 31 and 35 mg/L. The variation of the p25 value in each month was not significant, but the variation of the p75 value in each month was significant. The p75 value in November was the highest, whereas the p75 values in July and August were the lowest. The data demonstrated that the influent ammonia nitrogen concentration was low in summer. The influent ammonia nitrogen concentration never exceeded the designed value in each month. In July, the influent ammonia nitrogen concentration occasionally exceeded the designed value, and often exceeded the designed value in the other 11 months. Even in the period from September to December, the median value of influent ammonia nitrogen concentration was higher than the designed value. The designed value of influent ammonia nitrogen concentration was lower, and the appropriate value of influent ammonia nitrogen concentration should be set at about 40 mg/L, according to the actual situation. The low concentration of influent ammonia nitrogen in July and August was ascribed to the heavy summer rainfall, specifically, due to the mixture of rainwater. The ammonia nitrogen concentration of influent in other months, especially from October to December, was high because sewage from some enterprises did not meet “Wastewater quality standards for discharge to municipal sewers (GB/T 31962-2015)”.

### 3.3. Variations of Effluent Quality Indicators of the Sewage Treatment Plant

#### 3.3.1. Effluent COD_Cr_

**Figure 5:**
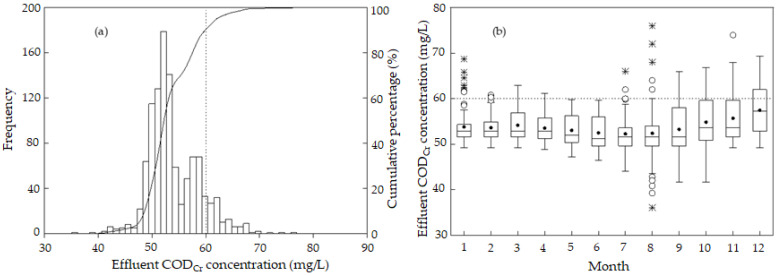
(**a**) Probability distribution and (**b**) monthly distribution of effluent COD_Cr_. The solid circle (•) represents the average. Outliers are marked by a hollow point (°) and extreme points are marked by an asterisk (*).

Figure 5 shows the monthly distribution and probability distribution of the effluent COD_Cr_ of the sewage treatment plant. Figure 5a shows the skewed distribution of effluent COD_Cr_. Effluent COD_Cr_ ranged from 36 to 76 mg/L and was mainly concentrated in the range of 51 to 53 mg/L. The cumulative percentage of the concentration below 60 mg/L, the First Level Class B standard value in “Discharge Standard of Pollutants for Municipal Wastewater Treatment Plants (GB18918-2002)”, was 89.4%. In other words, among all the measured data, 10.6% of the data exceeded the First Level Class B standard in “Discharge Standard of Pollutants for Municipal Wastewater Treatment Plants (GB18918-2002)”.

The lowest and highest effluent COD_Cr_ occurred in August (Figure 5b). The median value of effluent COD_Cr_ from January to November was between 51 and 53 mg/L, and the median value in December was the highest and reached 57.2 mg/L. The p75 value of effluent COD_Cr_ from January to November was below 60 mg/L, and the p75 value in December was higher than 60 mg/L. The frequencies of discrete points and extreme values were higher in July and August. The range of effluent COD_Cr_ from September to December was significant. The ranges of effluent COD_Cr_ in January, February, July, and August were small, indicating the constant effluent COD_Cr_. In May and June, effluent COD_Cr_ did not exceed 60 mg/L according to the First Level Class B standard value in the Urban Sewage Treatment Plant Factory Pollutant Discharge Standard. The p75 values of effluent COD_Cr_ in March, September, October, November, and December exceeded 60 mg/L, whereas only several data among all the effluent COD_Cr_ data in July and August exceeded 60 mg/L.

#### 3.3.2. Effluent Ammonia Nitrogen

**Figure 6:**
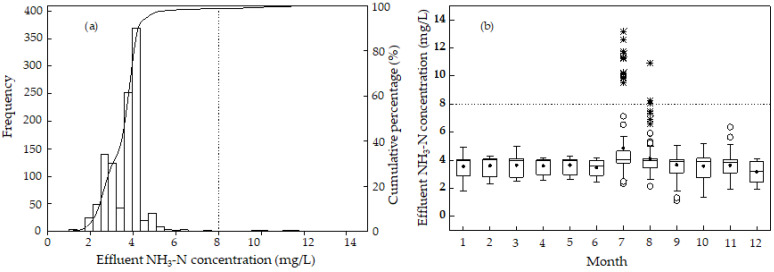
(**a**) Probability distribution and (**b**) monthly distribution of effluent NH_3_-N concentration. The solid circle (•) represents the average. Outliers are marked by a hollow point (°) and extreme points are marked by an asterisk (*).

Figure 6 demonstrates the monthly distribution and probability distribution of ammonia nitrogen concentration in the effluent of the sewage treatment plant. The range of ammonia nitrogen concentration in effluent was 1 to 13.16 mg/L, and the ammonia nitrogen concentration was mainly concentrated in the range of 4 to 4.5 mg/L (Figure 6a). The cumulative percentage of ammonia nitrogen concentration below 8 mg/L, the First Level Class B standard value in “Discharge Standard of Pollutants for Municipal Wastewater Treatment Plants (GB18918-2002)”, reached 98.7%. In other words, among all the measured data, only 1.3% of the data exceeded 8 mg/L, and these data were measured in July and August. The high effluent ammonia nitrogen concentration might be interpreted as follows (Figure 4 and Figure 6). The influent ammonia nitrogen concentration in July and August was generally low, and the treatment process of the sewage plant was normal. However, under the normal operation conditions, the occasionally high influent ammonia nitrogen concentration might result in it occasionally exceeding the standard value.

The median value of ammonia nitrogen concentration in effluent in December was low, and the median value in other months was approximately 4 mg/L (Figure 6b). In all the months except July and December, the median value was close to the p75 value. In July and August, extreme values were frequently found. These extreme values were the data exceeding the ammonia nitrogen concentration in effluent in the standard. As indicated in Figure 6a, the effluent ammonia nitrogen concentration exceeding the standard value is an extreme phenomenon (1.3%). In other words, under the normal state, the phenomenon that the effluent ammonia nitrogen concentration exceeded the standard value did not occur.

### 3.4. Correlation Analysis between the Indicators of Influent and Effluent

In this study, to determine the correlation between various indicators, Pearson’s correlation analysis was conducted among key wastewater indicators such as the daily quality and quantity of the influent and effluent of the sewage treatment plant (Table 2).

The influent COD_Cr_ concentration was negatively correlated with the influent ammonia nitrogen concentration. The probability that both influent COD_Cr_ concentration and influent ammonia nitrogen concentration were high simultaneously was extremely low. According to the analysis results in Section 3.2, 11.6% of the COD_Cr_ data of influent exceeded the designed value, 420 mg/L, and 41.4% of the ammonia nitrogen concentration data exceeded the designed value, 30 mg/L. Among all influent samples, the samples in which COD_Cr_ or ammonia nitrogen concentration exceeded the corresponding designed value accounted for 52.8%, whereas the samples in which both COD_Cr_ and ammonia nitrogen concentration exceeded the designed values accounted for only 0.1%.

Influent ammonia nitrogen concentration was significantly positively correlated with effluent COD_Cr_. When influent ammonia nitrogen concentration was high, effluent COD_Cr_ was high. The influent samples with the ammonia nitrogen concentration above the designed value, 30 mg/L, led to 94% of the effluent samples with COD_Cr_ exceeding the corresponding designed value. In other words, the higher effluent COD_Cr_ above the standard value was correlated with the high influent ammonia nitrogen concentration. The low C/N ratio in influent required an external carbon source to increase the effect of denitrification, which also caused the higher effluent COD_Cr_ above the standard value.

### 3.5. Average Daily Electricity Consumption (ADEC) and Electricity Consumption per Unit of Wastewater Quantity (UEC) Analysis

**Figure 7:**
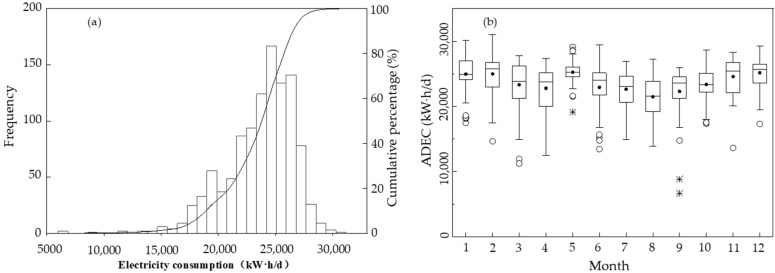
(**a**) Probability distribution and (**b**) monthly distribution of ADEC. The solid circle (•) represents the average. Outliers are marked by a hollow point (°) and extreme points are marked by an asterisk (*).

The average daily electricity consumption (ADEC) and electricity consumption per unit of wastewater quantity (UEC) of the sewage treatment plant (including the electricity consumption in the sewage treatment process and sludge dewatering) were examined in this study. Figure 7 demonstrates the monthly distribution and probability distribution of the electricity consumption of the sewage treatment plant.

Figure 7a shows that ADEC distribution belongs to a skewed distribution. ADEC ranged from 6660 kW·h/d to 31,020 kW·h/d. ADEC with the highest probability was 24,000 kW h/d, followed by 25,000 kW·h/d and 26,000 kW·h/d. The average of ADEC was 23,630 kW·h/d. ADEC was primarily distributed in the range of 17,000 to 28,000 kW·h/d. The cumulative percentage of ADEC below 28,000 kW·h/d was 98%.

The monthly ADEC was lowest in September and highest in February (Figure 7b). The median values and p75 values of ADEC in July, August, and September were lower. The median values and p75 values of ADEC in January, February, November, and December were higher. In other words, ADEC was low in summer and high in winter.

**Figure 8:**
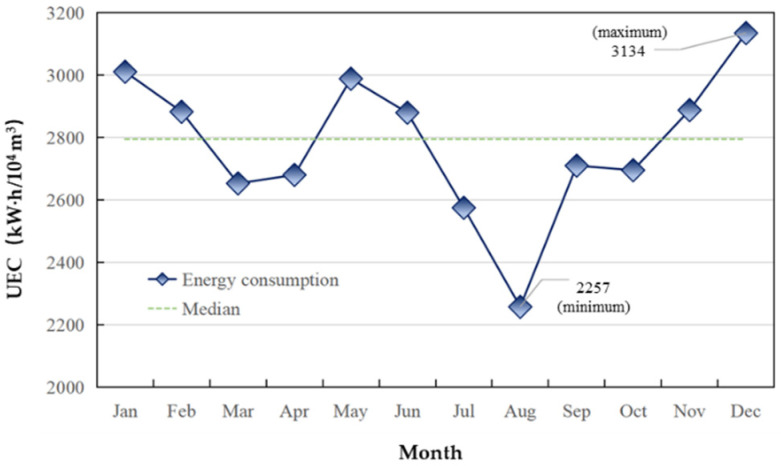
Monthly UEC.

The electricity consumption per unit wastewater quantity (UEC) is widely used to characterize the level of electricity consumption for the convenience of analysis. Figure 8 demonstrates the monthly UEC. The average monthly UEC was 2779 kW·h/10^4^ m^3^ (excluding electricity consumption for sludge treatment), which was close to or exceeded the average treatment efficiency of Chinese sewage treatment plants, 2800 kW·h/10^4^ m^3^ [46]. UEC was higher than the national average energy consumption in January, February, May, June, January, and December. UEC in winter for sewage treatment plants implementing the First Level Class B standard was relatively high, indicating that its energy consumption efficiency remained to be largely improved.

## 4. Discussion

The monthly and probability distributions of the influent and effluent indicators as well as the electricity consumption of the sewage treatment plant indicated that the quantity and quality of influent wastewater demonstrated complex dynamic trends. The range of influent quantity was large. The ranges of effluent COD_cr_ and ammonia nitrogen were small. To achieve the discharge standard with the sewage treatment process A^2^O, the corresponding energy consumption varied with the quality and quantity of influent wastewater. As a result, the electricity consumption was significantly affected by the quality and quantity of influent wastewater of the sewage treatment plant, as reported in previous studies [33,34,35,36,37]. In addition, influent wastewater quantity in summer was greater than that in winter. The concentration of influent wastewater pollutants in winter was greater than that in summer and ADEC and UEC in winter were greater than those in summer. Therefore, the relationships between electricity consumption and various factors (season, wastewater quantity and wastewater quality) were analyzed to determine the main factors affecting the energy consumption of urban sewage treatment plants.

### 4.1. Relationship between Electricity Consumption and Season

**Figure 9:**
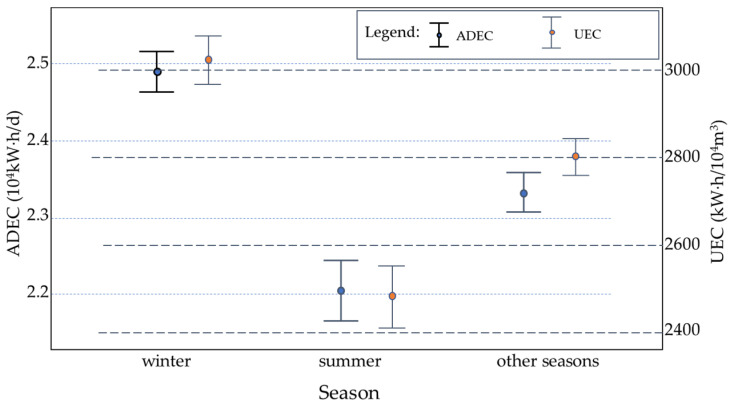
Relationship between electricity consumption and seasons.

**Figure 10:**
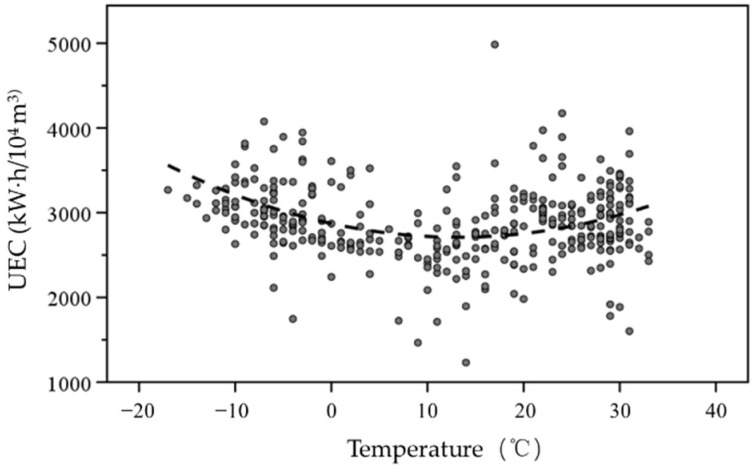
Regression diagram of temperature and electricity consumption per unit of wastewater.

According to the statistics of the electricity consumption of the sewage treatment plant in different seasons (winter, summer, and other seasons) (Figure 9), ADEC and UEC were higher in winter, lower in summer, and medium level in other seasons. The results indicated that the influence of season on the electricity consumption of the sewage treatment plant in Northeast China was significant. In cold winter, influent wastewater volume was small and pollutant concentrations were high, so the energy consumption of the plant was higher than national average energy consumption level. In summer influent wastewater quantity was higher and varied significantly and the pollutant concentrations were lower and scattered, so the energy consumption was close or lower than the national energy consumption level. In winter, especially when the temperature was low, the activity of activated sludge was significantly restricted and it was necessary to increase the time and amount of aeration to activate the activated sludge. The electricity consumption was negatively correlated with temperature below 0 °C (Figure 10). The lower the temperature was, the larger the electricity consumption was. The lower temperature had the more significant influence on electricity consumption, so the electricity consumption was high. Therefore, the influence of season (or temperature) on the energy consumption of the sewage treatment plant in Northeast China was significant.

### 4.2. Influences of the Wastewater Quantity and Quality on Electricity Consumption

In the study, the influences of influent wastewater quantity and quality on electricity consumption in summer scenario and winter scenario are, respectively, discussed below.

**Figure 11:**
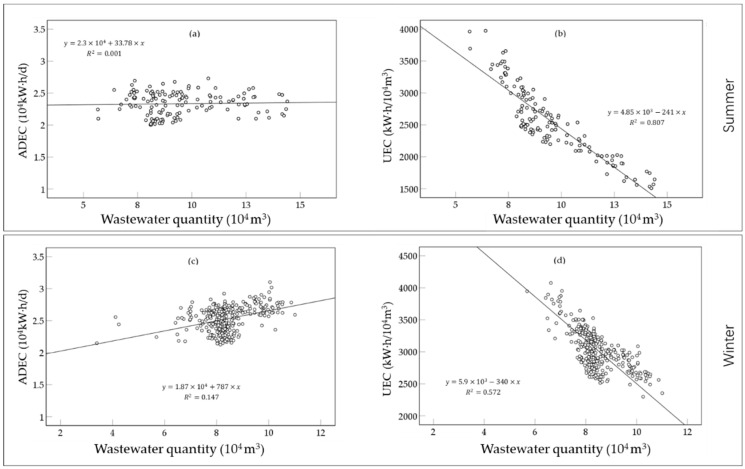
Variation of electricity consumption with wastewater quantity in different seasons. (Variation of (**a**) ADEC and (**b**) UEC in summer; Variation of (**c**) ADEC and (**d**) UEC in winter).

In summer, ADEC varied between 20,000 and 25,000 kW∙h/d, and ADEC slowly increased with wastewater quantity (Figure 11a). UEC was negatively correlated with wastewater quantity. UEC showed a decreasing trend with the increase in wastewater quantity, and the correction coefficient was −241 (Figure 11b). In winter, ADEC varied between 20,000 and 30,000 kW∙h/; ADEC showed a positive correlation with wastewater quantity and increased with the increase in wastewater quantity (Figure 11c). Like that in summer, UEC in winter was positively correlated with wastewater quantity, but the rate of reduction (the correlation coefficient was −340) was faster than that in summer (Figure 11d). Therefore, the influence of wastewater quantity on electricity consumption in winter was more significant than that in summer.

**Figure 12:**
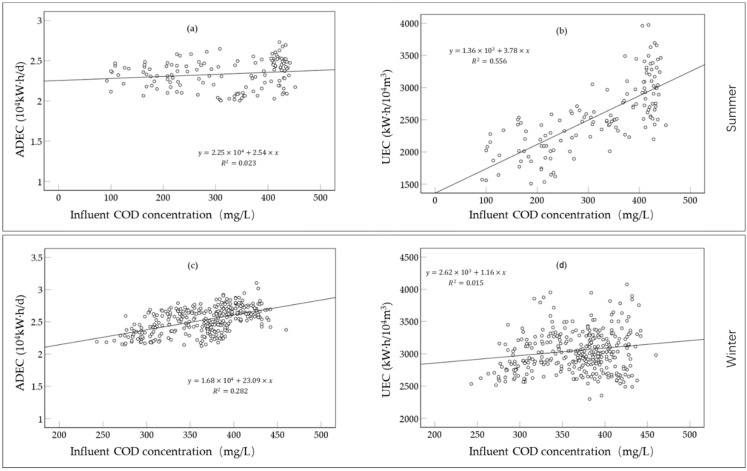
Variation of electricity consumption with influent wastewater COD concentration in different seasons. (Variation of (**a**) ADEC and (**b**) UEC in summer; Variation of (**c**) ADEC and (**d**) UEC in winter).

In summer, ADEC increased slightly with the increase in influent COD_cr_ concentration, and the regression coefficient was 2.54 (Figure 12a). UEC increased significantly with the increase in influent COD_cr_ concentration, and the regression coefficient was 3.78 (Figure 12b). In winter, the regression coefficient (23.09) of ADEC with influent COD_cr_ concentration was higher than that in the summer (Figure 12c), but the increase in UEC with influent COD_cr_ concentration was smaller than that in summer (Figure 12d). It could be concluded that the influence of pollutants on ADEC was greater in winter than in summer, and the influence of pollutants on UEC was greater in summer than in winter. This was because the summer rainwater entered the sewage treatment plant to dilute the sewage pollutants.

**Figure 13:**
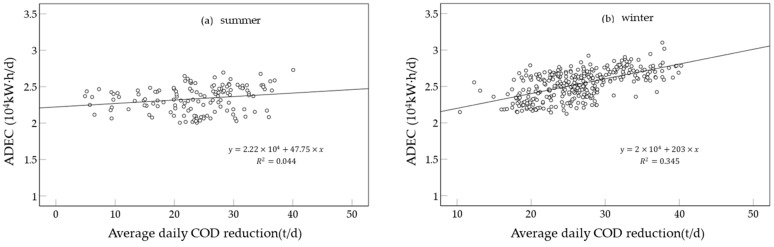
The variation of electricity consumption with the daily COD reduction in (**a**) summer and (**b**) winter.

In summer, ADEC was positively correlated with the average daily COD reduction. With the increase in the average daily COD reduction, ADEC showed a slight increase trend, displaying the growth slope of 47.75 (Figure 13a). In winter, ADEC also showed a positive correlation with average daily COD reduction. The regression coefficient (203) of ADEC with average daily COD reduction was greater than that in summer (Figure 13b). Therefore, the electricity consumption required for removing unit pollutants in winter was greater than that in summer.

Under normal circumstances, only domestic sewage and a small amount of industrial wastewater entered the sewage treatment plant in winter, and the wastewater quantity in winter was smaller than that in summer. In summer, due to the large and concentrated rainfall, partial rainwater entered the sewage treatment plant and resulted in its large influent wastewater quantity. Therefore, reducing the amount of rainwater entering the sewage treatment plant can reduce the wastewater quantity and pollutants, and the corresponding energy consumption can be reduced.

### 4.3. Influences of Abnormal Conditions on Electricity Consumption

In the above analysis, the wastewater quality indicators of some influent samples exceeded the corresponding designed values. In particular, the influent ammonia nitrogen concentration had two peaks. The second peak value was 1.37 times that of the designed value. Did this peak affect electricity consumption? The electricity consumption was examined below.

**Figure 14:**
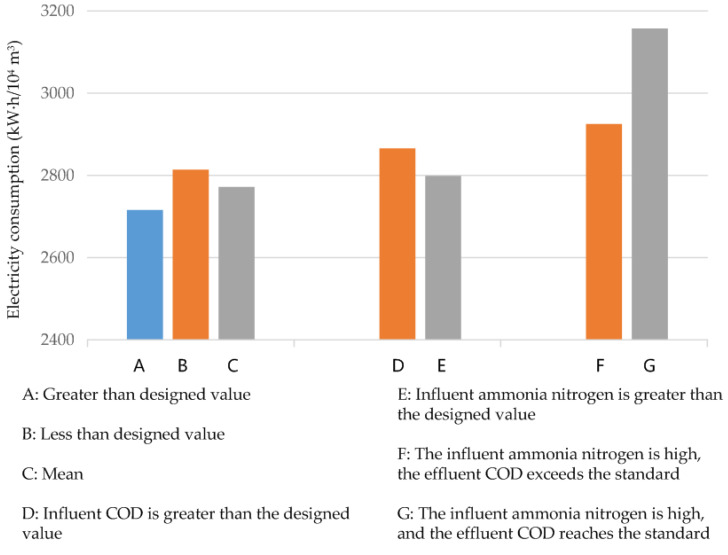
Diagram of electricity consumption per unit of wastewater.

With the quality and quantity indicators, influent wastewater samples could be classified into two categories. Firstly, in normal samples, the quantity and quality indicators were within corresponding designed ranges. Secondly, in abnormal samples, the quantity indicator or quality indicator exceeded the designed ranges. The abnormal samples could be further classified into two types: wastewater quantity exceeding the corresponding designed value and wastewater quality indicators exceeding the designed value. The wastewater quantity of the sewage treatment plant seldom exceeded the corresponding designed value, so only the scenario in which the wastewater quality indicators exceeded the designed value was examined. Two indicators (influent COD_Cr_ and influent ammonia nitrogen concentration) were, respectively, discussed below (Figure 14). Normal samples only accounted for 44.5%, whereas abnormal samples accounted for 55.5%. The average energy consumption was 2716 kW∙h/10^4^ m^3^ under normal conditions and 2814 kW∙h/10^4^ m^3^ under abnormal conditions. When influent ammonia nitrogen concentration was high and effluent COD_Cr_ exceeded the corresponding designed value, the average electricity consumption per unit of wastewater was 2925 kW∙h/10^4^ m^3^. However, when influent ammonia nitrogen concentration was high and effluent COD_Cr_ was lower than the corresponding designed value, the average electricity consumption per unit of wastewater was 3157 kW∙h/10^4^ m^3^. Under abnormal conditions, especially when the ammonia nitrogen concentration exceeded the designed value, the electricity consumption of the sewage treatment plant increased significantly.

## 5. Conclusions

In this study, a municipal wastewater treatment plant in Northeast China was selected as the research object, and the electricity consumption of the plant from 2017 to 2020 was analyzed with the multivariate statistical analysis methods, in order to explore the influences of the temperature and wastewater quantity and quality indicators of influent and effluent on energy consumption. The conclusion is as follows.

Firstly, in the investigation period, the range of influent quantity in the sewage treatment plant was larger in July and August and smaller in January, March, November, and December. The influent quantity in summer was larger because some rainwater entered the sewage treatment plant. In winter, the average daily electricity consumption was higher than that in summer. ADEC showed a positive correlation with wastewater quantity and the correlation coefficient in winter was larger than that in summer. UEC was negatively correlated with wastewater quantity and the correlation coefficient in winter was also larger than that in summer. Therefore, the influence of wastewater quantity on electricity consumption in winter was greater than that in summer. It is recommended that cities in Northeast China implement rainwater and sewage diversion, strengthen the operation and management of sewage treatment plants, and reduce the quantity of rainwater into sewage treatment plants, thereby reducing energy consumption.

Secondly, influent COD_Cr_ was the highest in December; lower in July, August, September, and October; and lowest in August. The influent ammonia nitrogen concentration was lowest in August and highest in November. Influent COD_Cr_ was negatively correlated with influent ammonia nitrogen concentration. Influent COD_Cr_ in 11.6% of the samples exceeded the corresponding designed value, and influent ammonia nitrogen concentration in 41.4% of the samples exceeded the corresponding designed value. Effluent COD_Cr_ in 10.6% of the samples exceeded the First Level Class B standard. In addition, unqualified COD_Cr_ in 94% of the effluent samples was ascribed to the unqualified ammonia nitrogen concentration in the influent samples. The probability of abnormal conditions was higher than that of normal conditions, and the electricity consumption level under abnormal conditions was higher than that under normal conditions. It is recommended that related departments strengthen management and reduce the discharge of wastewater from enterprises that do not meet the discharge standards.

Thirdly, in summer, ADEC was positively correlated with the average daily COD_Cr_ reduction. With the increase in the average daily COD_Cr_ reduction, ADEC showed a slightly increasing trend. In winter, ADEC also showed a positive correlation with average daily COD_Cr_ reduction. The correction coefficient of ADEC with average daily COD_Cr_ reduction was greater than that in summer. Therefore, the electricity consumption required for removing unit pollutants in winter was greater than that in summer. It is recommended that activated sludge microorganisms suitable for low temperature should be further explored and utilized.

Fourthly, average electricity consumption was close to that of Chinese national average energy consumption. ADEC was higher in winter, lower in summer, and medium in other seasons. In addition, when it was below 0 °C, the lower the temperature, the higher the electricity consumption. In Northeast China, the influences of seasons and temperatures on the electricity consumption of sewage plants were significant. It is recommended to take appropriate insulation measures in winter to reduce electricity consumption.

The study provides the technical and theoretical basis for the design, operation, energy reservation, and consumption reduction in sewage treatment plants in Northeast China.

## Figures and Tables

**Table 1:** Statistics indicators of a wastewater treatment plant in Northeast China.

Indicators	Minimum Values	Maximum Values	Means	Standard Deviations
Wastewater quantity (10^4^ m^3^/d)	0.9538	14.4031	8.531588	1.2101017
Electricity consumption (kWh/d)	6660	31,020	23,630.53	3031.561
Influent COD_Cr_ (mg/L)	92	460	354.69	64.348
Effluent COD_Cr_ (mg/L)	36.0	76.0	53.860	4.7255
Influent ammonia nitrogen (mg/L)	19.55	41.20	30.6987	4.53727
Ammonia nitrogen in effluent (mg/L)	1.12	13.16	3.7135	1.10612

**Table 2:** Pearson correlation analysis of various indicators of influent and effluent of the sewage treatment plant.

	Influent COD_Cr_	Effluent COD_Cr_	Influent Ammonia Nitrogen	Effluent Ammonia Nitrogen
Influent COD_Cr_	1			
Effluent COD_Cr_	−0.279 **	1		
Influent ammonia nitrogen	−0.440 **	0.551 **	1	
Effluent ammonia nitrogen	−0.025	−0.181 **	−0.290 **	1

** Significant correlation at the level of 0.01.

## Data Availability

The data presented in this study are available in the article.
